# Versatile Tunable Current-Mode Universal Biquadratic Filter Using MO-DVCCs and MOSFET-Based Electronic Resistors

**DOI:** 10.1155/2014/515348

**Published:** 2014-06-01

**Authors:** Hua-Pin Chen

**Affiliations:** Department of Electronic Engineering, Ming Chi University of Technology, Taiwan

## Abstract

This paper presents a versatile tunable current-mode universal biquadratic filter with four-input and three-output employing only two multioutput differential voltage current conveyors (MO-DVCCs), two grounded capacitors, and a well-known method for replacement of three grounded resistors by MOSFET-based electronic resistors. The proposed configuration exhibits high-output impedance which is important for easy cascading in the current-mode operations. The proposed circuit can be used as either a two-input three-output circuit or a three-input single-output circuit. In the operation of two-input three-output circuit, the bandpass, highpass, and bandreject filtering responses can be realized simultaneously while the allpass filtering response can be easily obtained by connecting appropriated output current directly without using additional stages. In the operation of three-input single-output circuit, all five generic filtering functions can be easily realized by selecting different three-input current signals. The filter permits orthogonal controllability of the quality factor and resonance angular frequency, and no inverting-type input current signals are imposed. All the passive and active sensitivities are low. Postlayout simulations were carried out to verify the functionality of the design.

## 1. Introduction

Since a low-voltage operating circuit becomes necessary, the current-mode technique is ideally suited for this purpose. Current-mode analog circuits have emerged in the implementation of analog functions. Current-mode active elements have been receiving considerable attention due to their potential advantages such as better linearity, wider bandwidth, larger dynamic range, and lower power consumption than the voltage-mode counterparts, for example, operational amplifiers [1, 2]. Simultaneously realized filter functions are applied in phase-locked loop frequency modulation stereo demodulators, touch-tone telephone tone decoders, and crossover networks used in three-way high-fidelity loudspeakers [3, 4].

Several single-input and multioutput current-mode universal biquadratic filters were presented in [5–12] but these configurations employ at least three current conveyors. In 2009, Horng et al. [13] proposed a novel current-mode universal filter with five inputs and two outputs using two multioutput second-generation current conveyors and five passive elements. However, one of the output currents is not at high impedance output terminal. In the same year, Yuce and Minaei [14] proposed another current-mode universal filter with a single input and three outputs. The resonance angular frequency (*ω*_*o*_) and the quality factor (*Q*) of the filter cannot be orthogonally controllable. In 2012, a versatile current-mode universal filter was proposed in [15]. This circuit employs three multioutput differential voltage current conveyors (MO-DVCCs). A current-mode universal biquadratic filter employing two plus-type dual current output terminals differential voltage current conveyors (DO-DVCCs), two grounded capacitors, and two grounded resistors was presented in [16]. The circuit can realize the lowpass (LP), bandpass (BP), highpass (HP), bandreject (BR), and allpass (AP) transfer functions by choosing the appropriate input and output terminals but it needs passive component-matching conditions. Moreover, the *ω*_*o*_ and *Q* parameters of the filter cannot be orthogonally controllable.

This paper presents a new and better versatile tunable current-mode universal filter employing only two MO-DVCCs. Either two-input three-output or three-input single-output current-mode universal biquadratic filter can work in the same configuration. The proposed circuit enjoys the following advantages: (i) the employment of only two MO-DVCCs, (ii) the employment of only grounded capacitors, (iii) the employment of MOSFET-based electronic resistors that act as three grounded resistors at *X* terminals of MO-DVCCs suitable for electronic tunability with control voltage applied to the gate and absorbing series parasitic resistances at *X* terminals of MO-DVCCs, (iv) simultaneous realization of BP, BR, and HP filtering responses with two-input and three-output while the AP filtering response can be easily obtained by connecting appropriated output current directly without using additional stages, (v) realization of LP, BP, HP, BR, and AP filtering responses with three-input and single-output by selecting different three-input current signals, (vi) high-output impedance good for cascade ability for the current-mode circuits, (vii) no capacitor is connected at the port *X* of the current conveyors, (viii) no need to employ inverting-type input current signals, (ix) orthogonal controllability of *ω*_*o*_ and *Q*, and (x) low active and passive sensitivity performances. To the best knowledge of the author, any versatile tunable current-mode universal biquad employing only two MO-DVCCs has not been reported in the literature earlier. In Table 1, the main features of the proposed new circuit are compared with recently reported DVCC-based current-mode universal function filters. It can be seen that the proposed circuit enjoys all the advantages of the recently reported in [15] and employs one fewer active component.

## 2. Circuit Description

**Figure 1:**
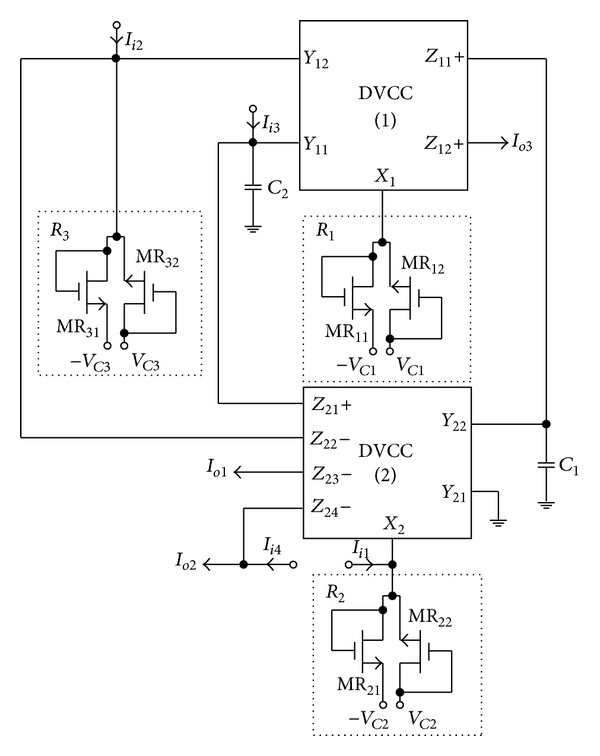
Proposed electronically tunable current-mode universal biquadratic filter.

As a current-mode active device, the DVCC has the advantages of both second-generation current conveyor (such as large signal bandwidth, great linearity, and wide dynamic range) and the differential difference amplifier (such as high input impedance and arithmetic operation capability) [17]. This element is a versatile building block whose applications exist in the literature [15–23]. The terminal characteristic of the DVCC can be given by *I*_*Y*1_ = *I*_*Y*2_ = 0, *V*_*X*_ = *V*_*Y*1_ − *V*_*Y*2_, *I*_*Z*+_ = *I*_*X*_, and *I*_*Z*−_ = −*I*_*X*_ [15–23]. The proposed versatile tunable current-mode universal biquadratic filter with high output impedance is shown in Figure 1. It is based on two MO-DVCCs, two grounded capacitors, and three grounded resistors by MOSFET-based electronic resistors. As shown in Figure 1, each resistor *R*_*i*_ (*i* = 1,2, 3) is realized by two MOS transistors (MR_*i*1_ and MR_*i*2_) that can be adjusted with their corresponding control voltages *V*_*Ci*_ and −*V*_*Ci*_. The two MOS transistors are operated in saturation region. The matched transistors MR_*i*1_ and MR_*i*2_ are diode connected. Using the square law characteristic, the drain currents in MR_*i*1_ and MR_*i*2_ can be expressed as [23, 24]

(1) $$\begin{array}{c} I_{di1}=\frac{\mu_{n}C_{ox}}{2}\frac{W}{L}{\left(V_{Ci}-V_{\text{in}}-V_{Tn}\right)}^{2}\\ I_{di2}=\frac{\mu_{n}C_{ox}}{2}\frac{W}{L}{\left(V_{\text{in}}-\left(-V_{Ci}\right)-V_{Tn}\right)}^{2}, \end{array}$$

where *μ*_*n*_ is carrier mobility, *C*_*ox*_ is the gate capacitance per unit area, *V*_*Tn*_ is the threshold voltage, and *W* and *L* are the channel length and width, respectively. A voltage *V*_in_ is applied to the central node of the electronic resistor circuit, developing a current *I*_in_ into the node. Using Kirchhoff's current law with (1), a simple algebraic expression can be obtained for the resistance as

(2) $$\begin{array}{c} R_{i}=\frac{V_{\text{in}}}{I_{\text{in}}}=\frac{V_{\text{in}}}{I_{di2}-I_{di1}}=\frac{L}{2\mu_{n}C_{ox}W\left(V_{Ci}-V_{Tn}\right)}. \end{array}$$

Equation (2) is applicable when both MOS transistors remain in the saturation region, which is true if |*V*_in_| < (*V*_*Ci*_ − *V*_*Tn*_). Straightforwardly analyzing the filter in Figure 1, the following three-output current signals can be derived as:

(3) $$\begin{array}{c} I_{o1}=\frac{1}{D\left(s\right)}\left[s^{2}C_{1}C_{2}R_{1}R_{2}I_{i1}-sC_{2}R_{3}I_{i2}+I_{i3}\right] \end{array}$$

(4) Io2=1D(s)[s2C1C2R1R(Ii1+Ii4)2−sC2R3(Ii2−Ii4)+(Ii3+Ii4)]

(5) Io3=1D(s)[−(s2C1C2R2R3+sC1R2)Ii1−s2C1C2R2R3Ii2+sC1R2Ii3]

*D*(*s*) is given by

(6) $$\begin{array}{c} D\left(s\right)=s^{2}C_{1}C_{2}R_{1}R_{2}^{}+sC_{2}R_{3}+1. \end{array}$$

Depending on the status of the biquad input four currents, *I*_*i*1_, *I*_*i*2_, *I*_*i*3_, and *I*_*i*4_, numerous filtering functions are obtained. There are two cases shown as follows.

**Case 1**

If *I*_*i*1_ = *I*_*i*3_ = 0 (opened) and *I*_*i*2_ = *I*_*i*4_ = *I*_in_ is given by the input current signals, then the following three current transfer functions are obtained:

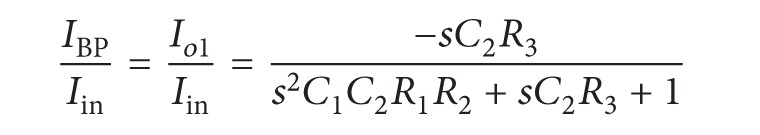
(7)

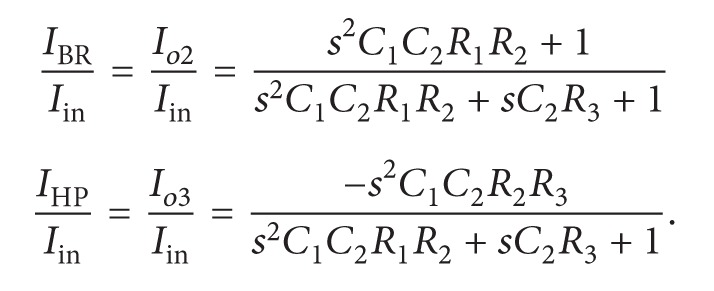
(8)
Thus, the circuit realizes an inverting BP response at *I*_*o*1_, a noninverting BR response at *I*_*o*2_, and an inverting HP signal at *I*_*o*3_. A current-mode AP response is easily obtained by connecting the two currents *I*_*o*1_ and *I*_*o*2_, yielding a transfer function as follows:

(9) $$\begin{array}{c} \frac{I_{\text{AP}}}{I_{\text{in}}}=\frac{I_{o1}+I_{o2}}{I_{\text{in}}}=\frac{s^{2}C_{1}C_{2}R_{1}R_{2}^{}-sC_{2}R_{3}+1}{s^{2}C_{1}C_{2}R_{1}R_{2}^{}+sC_{2}R_{3}+1}. \end{array}$$

Because the output impedances of the currents *I*_*o*1_, *I*_*o*2_, and *I*_*o*3_ are very high, the three-output terminals, *I*_*o*1_, *I*_*o*2_, and *I*_*o*3_, can be directly connected to the next stage, respectively. The two grounded resistors by MOSFET-based resistors are connected to the two *X* terminals of the two MO-DVCCs, respectively. This design offers another feature of a direct incorporation of the parasitic resistance at the *X* terminal of the MO-DVCC (*R*_*X*_) as a part of the main resistance. From (7) to (9), it can be seen that the proposed circuit is a two-input three-output CM multifunction biquadratic filter.

**Case 2**

The specializations of the numerator in (3) result in all standard filtering functions:

- if *I*_*i*1_ = *I*_*i*2_ = 0 (opened), and *I*_*i*3_ = *I*_in_ is given by the input current signal, an LP filter can be obtained at *I*_*o*1_
- if *I*_*i*1_ = *I*_*i*3_ = 0 (opened), and *I*_*i*2_ = *I*_in_ is given by the input current signal, a BP filter can be obtained at *I*_*o*1_
- if *I*_*i*2_ = *I*_*i*3_ = 0 (opened), and *I*_*i*1_ = *I*_in_ is given by the input current signal, a HP filter can be obtained at *I*_*o*1_
- if *I*_*i*2_ = 0 (opened), and *I*_*i*1_ = *I*_*i*3_ = *I*_in_ is given by the input current signal, a BR filter can be obtained at *I*_*o*1_
- if *I*_*i*1_ = *I*_*i*2_ = *I*_*i*3_ = *I*_in_ is given by the input current signal, an AP filter can be obtained at *I*_*o*1_.

From (3), it can be seen that the gain constants for the LP, BP, HP, BR, and AP responses are equal to unity in magnitude. The circuit does not need critical passive component-matching conditions and inverting-type current input signals to realize the above five generic filtering responses in the design. Obviously, it is a three-input and single-output universal current-mode biquadratic filter.

From the denominator polynomial of the transfer functions given in (6), the *ω*_*o*_ and *Q* of the filter are expressed as

(10) $$\begin{array}{c} \omega_{o}=\sqrt{\frac{1}{R_{1}R_{2}C_{1}C_{2}}}\\ Q=\frac{1}{R_{3}}\sqrt{\frac{R_{1}R_{2}C_{1}}{C_{2}}}. \end{array}$$

It should be noted that the parameter *Q* can be adjusted by changing the resistor *R*_3_ without disturbing the parameter *ω*_*o*_. Also, the passive *ω*_*o*_ and *Q* sensitivities are equal to or smaller than unity. Taking into account the nonidealities of a DVCC, namely, *V*_*X*_ = *β*_*a*_ *V*_*Y*1_ − *β*_*b*_ *V*_*Y*2_, *I*_*Z*+_ = *α*_*a*_ *I*_*X*_, and *I*_*Z*−_ = −*α*_*b*_ *I*_*X*_, where *α*_*m*_ = 1 − *ε*_*mi*_ (*m* = *a*, *b*) and *β*_*n*_ = 1 − *ε*_*nv*_ (*n* = *a*, *b*). Here *ε*_*mi*_(|*ε*_*mi*_| ≪ 1) and *ε*_*nv*_(|*ε*_*nv*_ | ≪1) represent the current and voltage tracking errors of the MO-DVCCs, respectively. The nonideal *ω*_*o*_ and *Q* are obtained by

(11) $$\begin{array}{c} \omega_{o}=\sqrt{\frac{\alpha_{a11}\alpha_{a21}\beta_{a11}\beta_{b22}}{R_{1}R_{2}C_{1}C_{2}}}\\ Q=\frac{1}{\alpha_{b22}\beta_{b12}R_{3}}\sqrt{\frac{\alpha_{a21}\beta_{a11}R_{1}R_{2}C_{1}}{\alpha_{a11}\beta_{b22}C_{2}}}. \end{array}$$

The active and passive sensitivities of the filter parameters *ω*_*o*_ and *Q* are shown as

(12)Sαa11,αa21,βa11,βb22ωo=−SR1,R2,C1,C2ωo=12(13)Sαa21,βa11Q=−Sαa11,βb22Q=SR1,R2,C1Q=−SC2Q=12Sαb22,βb12Q=SR3Q=−1,

which are equal to or less than unity in magnitude. Thus, the proposed filter of Figure 1 has small component sensitivities.

## 3. Simulation Results

**Figure 2:**
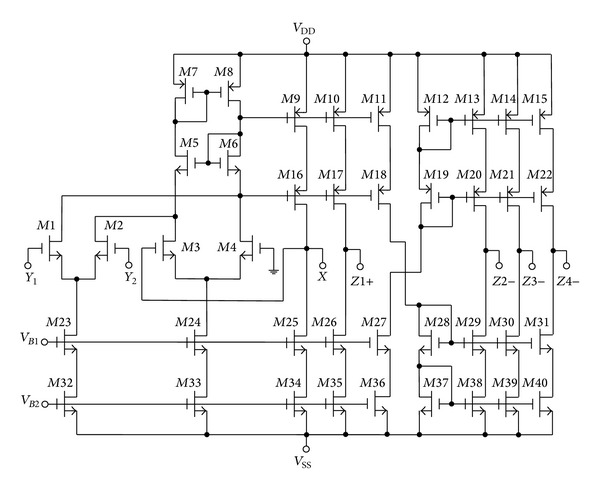
The CMOS implementation of the MO-DVCC.

**Figure 3:**
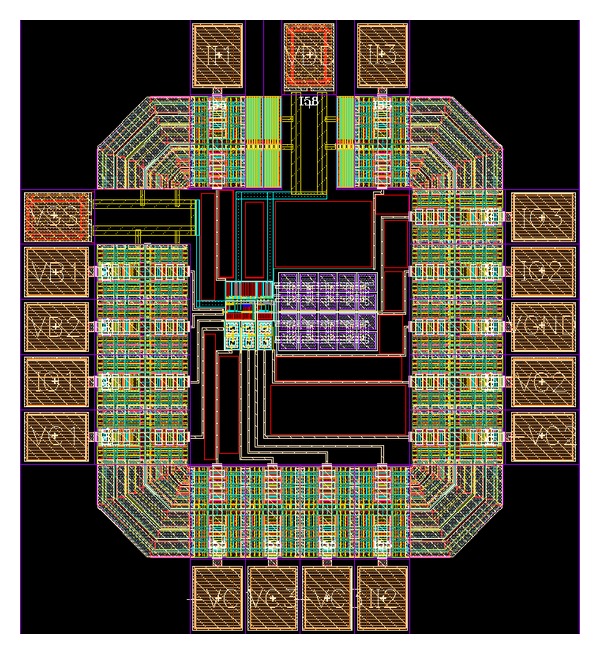
The chip layout of proposed current-mode universal biquadratic filter.

**Figure 4:**
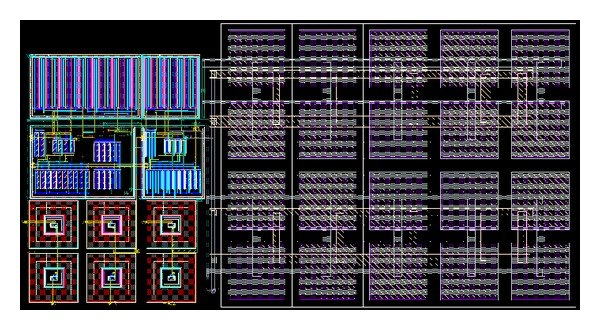
The core of the proposed current-mode universal biquadratic filter.

**Figure 5:**
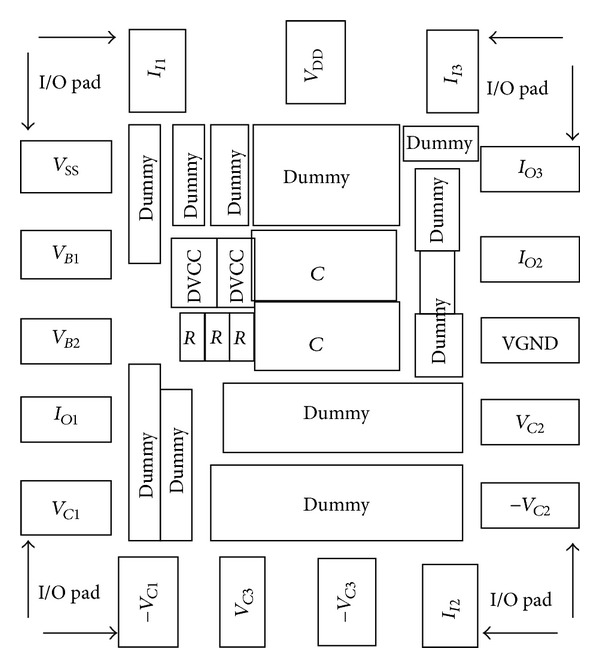
The layout floorplan.

**Figure 6:**
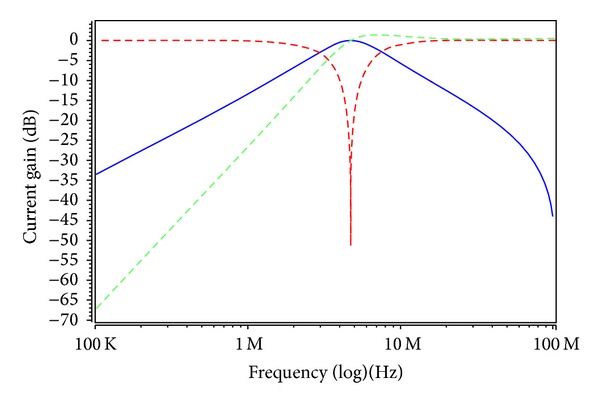
Postlayout simulation responses in Case 1 (highpass response: green; bandpass response: blue; bandreject response: red; and *V*_*C*_1__ = *V*_*C*2_ = *V*_*C*3_ = 1 V).

**Figure 7:**
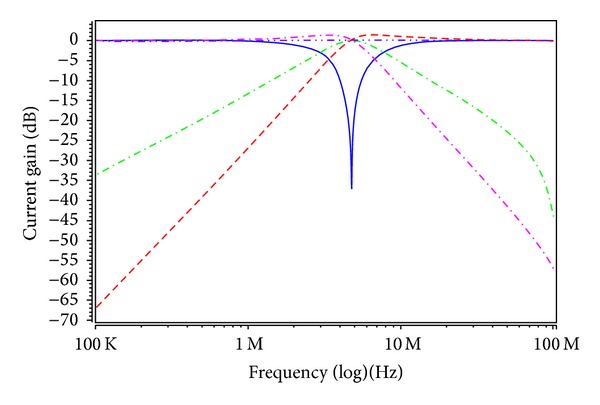
Postlayout simulation responses in Case 2 (highpass response: red; bandpass response: green; lowpass response: pink; bandreject response: blue; allpass response: purple; and *V*_*C*1_ = *V*_*C*2_ = *V*_*C*3_ = 1 V).

**Figure 8:**
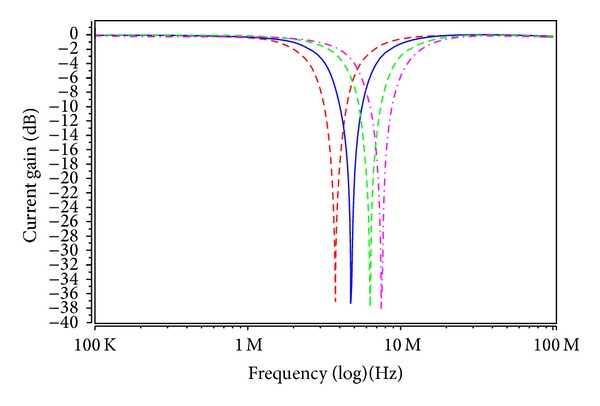
Electronic tunable of the BR frequency responses current-mode filter (*V*_*C*1_ = *V*_*C*2_ = *V*_*C*3_ = 0.95 V: red; *V*_*C*1_ = *V*_*C*2_ = *V*_*C*3_ = 1 V: blue; *V*_*C*1_ = *V*_*C*2_ = *V*_*C*3_ = 1.1 V: green; and *V*_*C*1_ = *V*_*C*2_ = *V*_*C*3_ = 1.2 V: pink).

**Figure 9:**
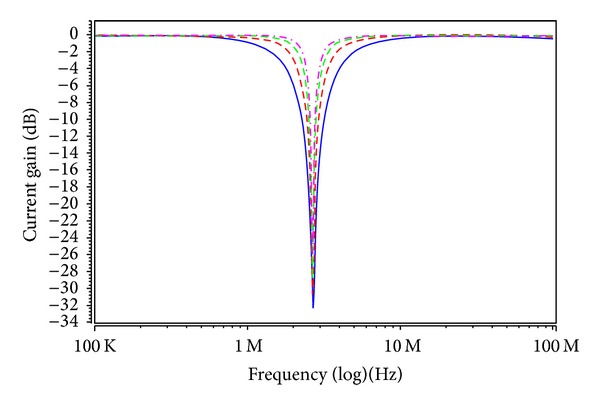
Electronic tunable of quality factor current-mode filter (*V*_*C*3_ = 0.9 V: blue; *V*_*C*3_ = 1 V: red; *V*_*C*3_ = 1.2 V: green; *V*_*C*3_ = 1.65 V: pink; and *V*_*C*1_ = *V*_*C*2_ = 0.9 V).

**Figure 10:**
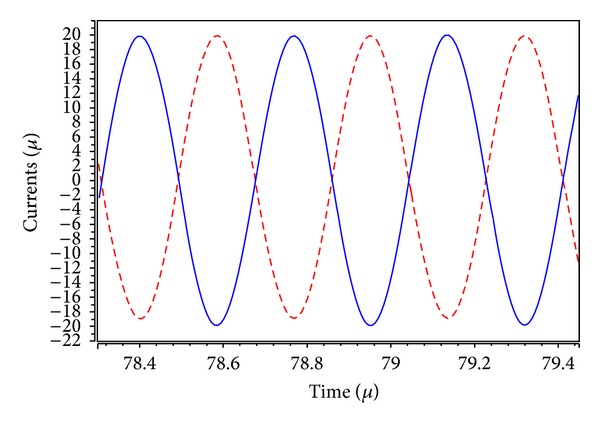
The input (blue line) and output (red line) waveforms of the BP response for a 2.72 MHz sinusoidal input current of 40 *μ*A (peak to peak).

To verify the theoretical study, the behavior of the proposed versatile tunable current-mode universal filter has been verified by using TSMC 0.18 *μ*m 1P6M CMOS process technology HSPICE simulations. The CMOS implementation of the MO-DVCC is shown in Figure 2 [15]. The dimensions of MOS transistors used in implementation of the MO-DVCC are given in Table 2. The supply voltages were *V*_DD_ = −*V*_SS_ = 0.9 V, and the biasing voltages were *V*_*B*1_ = −0.1 V and *V*_*B*2_ = −0.38 V. The layout of the entire schematic was done using Cadence's Virtuoso tool. Figures 3 and 4 show the overall chip layout and the detail layout of the filter core, respectively. The layout floorplan is shown in Figure 5 which explains elements placement. A design rules check (DRC) and a layout versus schematic (LVS) comparison were performed on the layout. The DRC checks for potential errors in the layout. The LVS checks the layout against the schematic and verifies that all the nets are matching. After the DRC and LVS were completed successfully, layout extraction was done. The extraction gives an overall idea about the parasitics of the design. All these processes are carried out using Cadence Virtuoso Schematic and Layout editor tool. The postlayout simulations were carried out to check the functionality of the design. The dimensions of the NMOS 3 V transistors MR_*i*1_ and MR_*i*2_ (*i* = 1,2, 3) in the proposed filter of Figure 3 are selected as *W*/*L* = 0.5 *μ*m/0.5 *μ*m and *C*_1_ = *C*_2_ = 3 pF. Different control voltages of *V*_*Ci*_ = 0.9, 0.95, 1, 1.1, 1.2, and 1.65 V for the MOSFET-based electronic resistor values 19.54, 13.86, 10.97, 8.24, 6.95, and 4.96 kΩ, respectively. Figures 6 and 7 show the current-mode postsimulated results at a resonance frequency of *f*_*o*_ ≈ 4.76 MHz and a quality factor of *Q* = 1 with *V*_*C*1_ = *V*_*C*2_ = *V*_*C*3_ = 1 V in Cases 1 and 2, respectively. In Figure 7, the magnitude of input currents *I*_*i*1_, *I*_*i*2_, and *I*_*i*3_ can be chosen as in Table 3. Showing the frequency tunability of the proposed filter by selecting equal control voltages of *V*_*C*1_ = *V*_*C*2_ = *V*_*C*3_ with different values of 0.95 V, 1 V, 1.1 V, and 1.2 V results in filter responses with a quality factor of *Q* = 1 and resonance frequencies of *f*_*o*_ ≈ 3.78, 4.76, 6.32, and 7.47 MHz, respectively. Thus we obtain different resonance frequencies at *I*_*o*1_ output terminal with *I*_*i*2_ = 0 and *I*_*i*1_ = *I*_*i*3_ = *I*_in_ for the BR responses as shown in Figure 8. To show the quality factor tunability of the proposed filter, by keeping the values of *V*_*C*1_ = *V*_*C*2_ = 0.9 V and varying only *V*_*C*3_, the *Q* can be controlled by the following setting: *V*_*C*3_ = 0.9 V to realize *Q* = 1, *V*_*C*3_ = 1 V to realize *Q* = 1.78, *V*_*C*3_ = 1.2 V to realize *Q* = 2.81, and *V*_*C*3_ = 1.65 V to realize *Q* = 3.94. Thus we obtain different *Q*s at *I*_*o*1_ output terminal with *I*_*i*2_ = 0 and *I*_*i*1_ = *I*_*i*3_ = *I*_in_ for the BR responses as shown in Figure 9. It is observed from Figures 6–9 that the filter postlayout simulation performs all the filter functions well, but the small departures filter responses mainly stems from the parasitic impedance effects and nonideal gains of MO-DVCC. To test the input dynamic range of the filter, the postlayout simulation has been repeated for a sinusoidal input signal at *f*_*o*_ = 2.72 MHz.  Figure 10 shows the input dynamic range of the inverting BP response at *I*_*o*1_ output terminal with *V*_*C*1_ = *V*_*C*2_ = *V*_*C*3_ = 0.9 V, *I*_*i*1_ = *I*_*i*3_ = 0, and *I*_*i*2_ = *I*_in_, which extends up to amplitude of 40 *μ*A (peak to peak) without signification distortion. The total power dissipation is found to be 0.438 mW. The chip area without pads is only 167.5 × 85.76 *μ*m^2^.

## 4. Conclusion

In 2012, a versatile current-mode universal filter was proposed in [15]. This circuit employs three MO-DVCCs. In this paper, the author also presents a versatile current-mode universal biquadratic filter. The new circuit employs only two MO-DVCCs, two grounded capacitors, and three grounded resistors by MOSFET-based electronic resistors. Either a two-input and three-output circuit or a three-input and single-output circuit current-mode universal biquadratic filter can work in the same configuration. The proposed filter can be used for realizing all of the standard filtering functions without inverting-type current input signals. The parameters *ω*_*o*_ and *Q* are orthogonally controllable. The postlayout simulations were carried out to verify the functionality of the design. The filter chip operates to 7.47 MHz and the power dissipation is 0.438 mW. The specifications of our analog filter chip are summarized in Table 4.

## Figures and Tables

**Table 1:** Comparison with recently reported of DVCC-based current-mode universal function filters.

Parameter	This work	[15]	[16]
Technology	CMOS 0.18 *μ*m	CMOS 0.18 *μ*m	CMOS 0.5 *μ*m
Power supply	±0.9 V	±0.9 V	±2.5 V
Power consumption	0.438 mW	0.462 mW	—
Number of active devices	2 MO-DVCCs	3 MO-DVCCs	2 DO-DVCCs
Simultaneous outputs	3	3	2
Availability of universal filter responses	Yes	Yes	Yes
Simultaneous realization of BP, HP, and BR filter responses	Yes	Yes	No
Orthogonal tunability of *ω*_*o*_ and *Q*	Yes	Yes	No
No matching condition required	Yes	Yes	No
Only grounded passive components used	Yes	Yes	Yes
Output current at high-output impedance	Yes	Yes	Yes

**Table 2:** The aspect ratios of the CMOS transistors in MO-DVCC implementation.

Transistors	*L* (*μ*m)	*W* (*μ*m)
M1–M4	0.36	3.6
M5-M6	0.36	7.2
M7–M22	0.18	14.4
M23–M40	0.36	5.4

**Table 3:** The *I*_*i*1_, *I*_*i*2_, and *I*_*i*3_ values selection for each filter function response.

Filter responses	Input selections		
*I*_*o*1_	*I*_*i*1_	*I*_*i*2_	*I*_*i*3_
LP	0	0	*I*_in_
BP	0	*I*_in_	0
HP	*I*_in_	0	0
BR	*I*_in_	0	*I*_in_
AP	*I*_in_	*I*_in_	*I*_in_

**Table 4:** Summary of performance.

Process technology	TSMC 0.18 *μ*m 1P6M CMOS
Power supply	±0.9 V
Filter mode	Current-mode
Filter responses	LP, BP, HP, BR, and AP
Operation frequency	7.47 MHz
Power dissipation	0.438 mW
Core area	167.5 × 85.76 *μ*m^2^
